# The mitochondrial genome structure of *Xenoturbella bocki *(phylum Xenoturbellida) is ancestral within the deuterostomes

**DOI:** 10.1186/1471-2148-9-107

**Published:** 2009-05-18

**Authors:** Sarah J Bourlat, Omar Rota-Stabelli, Robert Lanfear, Maximilian J Telford

**Affiliations:** 1 Department of Invertebrate Zoology, Swedish Museum of Natural History, Box 50007, SE-104 05 Stockholm, Sweden; 2 Department of Genetics, Evolution and Environment, University College London, Darwin Building, Gower Street, London WC1E 6BT, UK; 3 Current address: Centre for Macroevolution and Macroecology, School of Biology, Australian National University, Canberra ACT 0200, Australia

## Abstract

**Background:**

Mitochondrial genome comparisons contribute in multiple ways when inferring animal relationships. As well as primary sequence data, rare genomic changes such as gene order, shared gene boundaries and genetic code changes, which are unlikely to have arisen through convergent evolution, are useful tools in resolving deep phylogenies. *Xenoturbella bocki *is a morphologically simple benthic marine worm recently found to belong among the deuterostomes. Here we present analyses comparing the *Xenoturbella bocki *mitochondrial gene order, genetic code and control region to those of other metazoan groups.

**Results:**

The complete mitochondrial genome sequence of *Xenoturbella bocki *was determined. The gene order is most similar to that of the chordates and the hemichordates, indicating that this conserved mitochondrial gene order might be ancestral to the deuterostome clade. Using data from all phyla of deuterostomes, we infer the ancestral mitochondrial gene order for this clade. Using inversion and breakpoint analyses of metazoan mitochondrial genomes, we test conflicting hypotheses for the phylogenetic placement of *Xenoturbella *and find a closer affinity to the hemichordates than to other metazoan groups. Comparative analyses of the control region reveal similarities in the transcription initiation and termination sites and origin of replication of *Xenoturbella *with those of the vertebrates. Phylogenetic analyses of the mitochondrial sequence indicate a weakly supported placement as a basal deuterostome, a result that may be the effect of compositional bias.

**Conclusion:**

The mitochondrial genome of *Xenoturbella bocki *has a very conserved gene arrangement in the deuterostome group, strikingly similar to that of the hemichordates and the chordates, and thus to the ancestral deuterostome gene order. Similarity to the hemichordates in particular is suggested by inversion and breakpoint analysis. Finally, while phylogenetic analyses of the mitochondrial sequences support a basal deuterostome placement, support for this decreases with the use of more sophisticated models of sequence evolution.

## Background

Mitochondria have evolved from eubacterial endosymbionts related to the α-proteobacteria [1]. The primitive state for the mitochondrial genome probably resembled that found in the protozoan *Reclinomonas americana*, which has a 69,034 bp genome which still retains eubacterial features of genome organisation such as operons [2]. During their evolutionary history, many mitochondrial genomes have been reduced in size, having lost many genes, some of which have been transferred to the nucleus. As a result, mitochondrial genomes vary widely in size and structure amongst animals, plants, fungi and protists. Within the Metazoa, however, mitochondrial genomes show surprising conservation of size and composition [3], almost invariably containing 13 protein coding genes, 2 ribosomal genes and 22 tRNAs, necessary for the transcription of the mitochondrially encoded genes. The order of those genes on the mitochondrial genome differs widely among the metazoan phyla, but can also show surprising conservation in organisation within specific metazoan clades, such as the Vertebrata [4] and the Ecdysozoa [5]. This striking conservation in gene order and composition observed between certain animal phyla is considered too complex to have arisen in any way other than by common ancestry and can be a powerful tool for resolving animal relationships. Breakpoint and maximum parsimony analyses have revealed phylogenetic signal derived from the mitochondrial gene order allowing inference of evolutionary relationships among the metazoan phyla, and support aspects of the new animal phylogeny (Lophotrochozoa/Ecdysozoa) [6].

The deuterostomes are a monophyletic group of animals comprised of the chordates (vertebrates, cephalochordates and urochordates), the echinoderms, the hemichordates and the recently included xenoturbellids [7,8]. Previous morphological studies suggest alternative placements for *Xenoturbella*, as a basal bilaterian [9], a turbellarian flatworm [10], a sister group to the echinoderms or hemichordates [11,12] or a bivalve mollusc [13,14]. Recent molecular studies based primarily on nuclear genes seem to favour a placement as a sister group to the Ambulacraria (echinoderms + hemichordates) [7,8,15]. Conversely, mitochondrial sequences support a basal deuterostome position [16], but when amino acids with a different genetic code are excluded, *Xenoturbella *was recovered as basal ambulacrarian [7].

Traditional evolutionary morphological hypotheses united the deuterostome phyla (other than Xenoturbellida) on the basis of radial cleavage of the embryo and of deuterostomy, in which the anus rather than the mouth develops from the blastopore during early embryonic development. This grouping has largely remained robust to molecular phylogenetic analyses (although chaetognaths and lophophorates are no longer considered deuterostomes), as opposed to the protostomes in which many groupings based on morphological similarities have not been supported by molecular data. Current molecular phylogenetic analyses place the echinoderms and the hemichordates together in a group called the Ambulacraria, and the chordates (vertebrates, urochordates and cephalochordates) as their monophyletic sister group [7,17,18].

Phylogenetic studies of 18s rRNA sequences, expressed sequence tags (ESTs) and mitochondrial genome data have all suggested that the benthic marine worm *Xenoturbella *is placed in its own phylum, at the base of the Ambulacraria [8,7,15]. This is somewhat surprising in morphological terms, as *Xenoturbella *has no coelomic cavities, nor a through gut as in all the other members of the deuterostomes. It also has no other organs, no visible gonads nor centralized nervous system [10]. The most obvious explanation for this apparent simplicity might be that this animal is highly derived, and secondarily simplified. Other members of the deuterostome group, such as the echinoderms, can also be described as highly morphologically derived: they have a unique five-fold symmetry while all other deuterostomes are bilaterally symmetrical. All extant echinoderms also lack gill slits [19]. In addition to their highly derived morphology, the mitochondrial genomes of echinoderms are fast evolving and their gene order and genetic code varies both within and between echinoderm classes [20]. In contrast, and rather strikingly, the mitochondrial genomes of the vertebrates, hemichordates and *Xenoturbella bocki *are very similar in their gene order [21]. This conserved mitochondrial arrangement in 3 out of 4 deuterostome phyla is an indication that it is likely to represent the ancestral state of the deuterostome mitochondrial gene arrangement. Amongst the protostomes and diploblasts, mitochondrial gene orders appear to be significantly more variable [22]. Another study of *Xenoturbella*'s mitochondrial genome suggested that this conserved gene arrangement supports a basal position for *Xenoturbella *within the deuterostomes, and possibly among the Bilateria [16]. But until suitably conserved outgroups are found at the base of the Bilateria, it will be difficult to infer the ancestral bilaterian mitochondrial gene order. Recent advances in metazoan phylogenomics [15], will no doubt help in finding suitable outgroups at the base of the Bilateria for comparison. The full mitochondrial genomes of acoels and nemertodermatids, two phyla thought to be the most basal Bilateria, will be of particular interest in determining the composition of the ancestral bilaterian mitochondrial gene order. Partial mitochondrial genome sequences for the Nemertodermatid *Nemertoderma westbladi *and the acoel *Paratomella rubra *already indicate that the gene order in these groups is quite divergent from the ancestral deuterostome arrangement, and bears no similarity to that of other metazoan groups [23].

An interesting feature of mitochondrial DNA (mt DNA) is the control region, which is involved in transcription and replication of the heavy (H) strand and mayform a stable stem-loop structure [4]. The control region is called the D-loop region in vertebrates and its name comes from the nascent Heavy (H) strand that displaces the parental H strand and forms a typical D shaped structure [24]. Conserved structural features in the control region of mtDNA have been used in evolutionary studies [25,26] and have been extensively characterised in vertebrates [27], providing a useful comparison point to the non-vertebrate deuterostome groups including *Xenoturbella*.

Mitochondrial DNA primary sequences have also been extensively used for the inference of metazoan phylogeny, in particular in mammals and other deuterostome groups [28,20,16]. However, phylogenetic inference from mitochondrial sequences may be complicated by a number of misleading factors such as compositional heterogeneity [29], strand asymmetry [30] and accelerated substitution rates, which violate the assumptions of the commonly used models of evolution. A useful approach to overcome these problems is to improve the models of evolution used to infer phylogeny from these sequences. In particular more reliable empirical replacement matrices [31], and models that account for heterogeneities across sites [32] and among lineages [33] may significantly improve the fit of data to the model and therefore provide more reliable phylogenetic estimates.

In this study, we describe the gene order, composition and non-coding regions of the mitochondrial genome of *Xenoturbella bocki*. We infer the ancestral state of the mitochondrial gene order in the deuterostome common ancestor. Using breakpoint and inversion analysis, we test conflicting hypotheses of *Xenoturbella*'s phylogenetic position in a tree of the Metazoa and finally we use more sophisticated models of amino acid substitution to infer the phylogenetic position of *Xenoturbella *using mitochondrial amino acid sequence data.

## Results and discussion

### Mitochondrial genome structure

The mitochondrial genome of *Xenoturbella bocki *is a 15,234 bp circular DNA molecule, and has an A+T content of 64.48%. As in most other bilaterian mitochondrial genomes, there are 13 protein coding genes, 22 transfer RNAs and 2 ribosomal RNAs (*rrnS *and *rrnL*). ATG is used as a start codon for all of the protein coding genes, except *cox2*, which starts with GTG. *atp6*, *cox3*, *nd3 *and *nd4 *end on TA and *cox2 *on T, while all other genes end on the usual TAA. These attenuated stop codons are presumably completed by polyadenylation [34,35] (See additional file 1).

**Figure 1:**
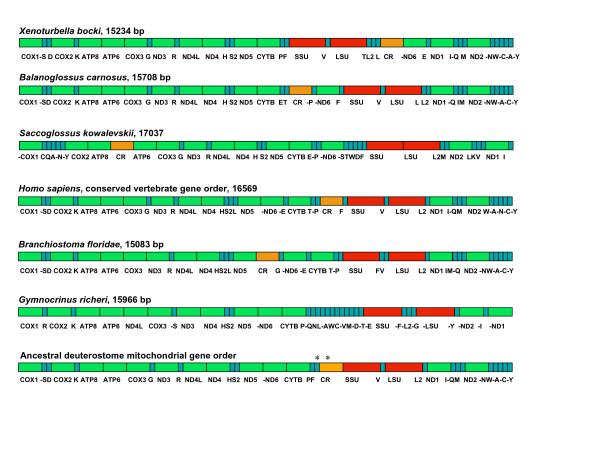
**Gene map of the *Xenoturbella bocki *mitochondrial genome compared to other deuterostome mitochondrial genomes**. *Xenoturbella bocki *gene order is compared to *Balanoglossus carnosus *and *Saccoglossus kowalevskii *(two hemichordates), *Homo sapiens, Branchiostoma floridae *(a cephalochordate) and *Gymnocrinus richeri *(a crinoid echinoderm). Below: Mitochondrial gene order reconstruction for the deuterostome common ancestor. *Position of the control region is uncertain. The most parsimonious scenario for the ancestral deuterostome was reconstructed using gene order information from species in the echinoderm, hemichordate, *Xenoturbella*, vertebrate and cephalochordate lineages. The urochordate lineage was too derived.

The gene order of the *Xenoturbella bocki *mitochondrial genome is shown in figure 1. Within the deuterostome group, the gene order of *Xenoturbella bocki *is most similar to that of the conserved vertebrate gene order as well as the hemichordate *Balanoglossus carnosus*, which has virtually the same gene order as the vertebrates [21]. In *Xenoturbella, Balanoglossus *and the vertebrates all protein coding genes follow the same order on the plus strand except for the control region and -*nd6*, the only protein coding gene on the minus strand. In the remaining deuterostome phyla, the echinoderms and the urochordates, the gene orders are very different, and vary extensively even amongst the echinoderm classes [20] (figure 1). Given that the echinoderm lineage is very derived, and considering the phylogenetic position of *Xenoturbella *as a separate phylum at the base of Ambulacraria [7,8], it is likely that the vertebrate/hemichordate/*Xenoturbella *arrangement represents the ancestral gene order for the deuterostome clade.

There are few gaps between the genes, except for a 40 bp gap between *trnE *and *nd1 *and a 334 bp gap between *trnL *and -*nd6*, which is likely to correspond to the control region for the heavy (H) strand and presumably forms a hairpin loop (additional file 1). There are a number of gene overlaps, the most significant being a 19 bp overlap between the end of *atp8 *and the start of *atp6 *on the same strand but translated in different reading frames (additional file 1). *atp8 *and *atp6 *also overlap in other metazoans, and are probably transcribed together. Another 6 bp overlap is present between the end of *nd4l *and the start of *nd4*. This overlap is also present in the vertebrate, hagfish, and *Saccoglossus kowalevskii *mitochondrial genomes. Even though these genes are adjacent in the cephalochordates *Branchiostoma sp*. and *Epigonichthys sp*., and in the hemichordate *Balanoglossus carnosus*, there is no overlap. In the more derived echinoderm and urochordate mitochondrial genomes, these genes are not adjacent. The proximity of these genes in *Xenoturbella*, Hemichordata, Vertebrata and Cephalochordata indicates that these genes may have been adjacent in the ancestral deuterostome mitochondrion although it does not allow us to know whether they overlapped or not. The presence of this 6 bp overlap between the end of *nd4l *and the start of *nd4 *in other phyla outside the deuterostomes (in the priapulid *Priapulus caudatus*, the brachiopod *Terebratulina retusa *and the annelid *Platynereis dumerilii*) indicates that this is likely to be an ancestral feature of bilterian genomes.

There are a number of overlaps in the genes coding for tRNAs: *trnH *overlaps with *trnS2 *by 6 bp and *trnS2 *overlaps with *nd5 *by 12 bp. Other tRNA genes which appear to be overlapping by 1 to 2 bp at the 3' end with another gene or tRNA may in fact be lacking these bases. The overlapping base is in all cases an A and could be later added to the tRNA by polyadenylation [36,37].

### Genetic code changes

Shared mitochondrial genetic codes changes between phyla are rare and complex events and can be used to infer common ancestry [38].*Xenoturbella bocki *has the standard invertebrate mitochondrial genetic code. It does not share the ATA codon change from methionine (M) to isoleucine (I) found in echinoderms and hemichordates. There is one further genetic code change found in echinoderms: AAA codes for asparagine (N) instead of lysine (K) [21]. In the hemichordate *Balanoglossus carnosus*, the codon AAA is absent however AAA codes for lysine in Saccoglossus and this is therefore an echinoderm specific change [21]. In *Xenoturbella bocki*, the AAA codon codes for lysine as in most other invertebrates (additional file 2). The genetic coding of ATA = I shared by echinoderms and hemichordates is an Ambulacrarian synapomorphy that is lacking in *Xenoturbella *and suggests that Xenoturbellida represents an independent lineage outside of the Ambulacraria.

### Reconstructing the ancestral deuterostome mitochondrial gene order

By comparing the gene boundaries found in the mitochondrial genomes of *Xenoturbella bocki*, Hemichordata (*Balanoglossus carnosus *and *Saccoglossus kowalevskii*), Vertebrata (using *Homo sapiens*, which has the conserved vertebrate gene order), Cephalochordata (*Branchiostoma floridae*) and Echinodermata (*Gymnocrinus richeri*), we inferred the ancestral deuterostome mitochondrial gene order (figure 1).

We find that in *Xenoturbella *and the two hemichordates, *Saccoglossus kowalevskii *and *Balanoglossus carnosus, nd5 *and *cob *are adjacent. While the *nd5*, *cob *gene boundary is common to *Xenoturbella *and the hemichordates, the final location of *nd6*/*trnE *(which is present in between *nd5 *and *cob *in the chordates) is different in the two clades suggesting this could be a parallel change. Alternatively, there could have been an additional translocation event in the lineage leading to *Xenoturbella*. If the *nd5, -nd6*, *trnE*, *cob *gene order found in non-avian vertebrates is to represent the ancestral state, the *nd5*, -*nd6*, *cob *gene arrangement found in 15 out of 20 of the sequenced echinoderm mitochondrial genomes could represent an intermediate step in the lineage leading to the *Xenoturbella and *hemichordate gene order (*nd5, cob*). More mitochondrial genomes within the hemichordates and especially from the basal pterobranch hemichordates will allow us to establish whether the *nd5*, -*nd6, cob *arrangement or *nd5, cob *represents the ancestral state.

Gene rearrangements have been shown to be more prevalent around the control region, indicating that certain constraints operate on mitochondrial rearrangements, and the proximity of *nd5, nd6 *and *cob *to the control region may have resulted in convergent gene translocations. Interestingly, the *nd5, cob *gene order is also found in birds [39], probably through a convergent change.

To reconstruct the gene order of the deuterostome common ancestor (figure 1), we used parsimony reconstruction, based on comparisons of the genomes of *Xenoturbella bocki*, *Balanoglossus carnosus, Saccoglossus kowalevskii*, *Branchiostoma floridae*, the conserved non avian-vertebrate gene order, and the crinoid *Gymnocrinus richeri*, a basal echinoderm [40]. Where a consensus could not be reached, the primitive state was determined by looking at mitochondrial genomes within the protostomes. Minimum breakpoint reconstruction of the mitochondrial gene order for the ancestor of all bilaterians shows that this gene order is virtually identical to that inferred by us for the deuterostome ancestor, with a few differences in the position of tRNAs [6]. It has previously been argued that this ancestral gene order might be uninformative in terms of making assumptions about deuterostome monophyly, or even about *Xenoturbella *deuterostome affinities [16]. To test this assertion, we compared the relative scores obtained by breakpoint and inversion analysis of alternative positions of *Xenoturbella *among the Bilateria by using a large set of metazoan mitochondrial genomes including a large number of deuterostomes.

### Inversion and breakpoint analysis

To test alternative hypotheses of *Xenoturbella*'s phylogenetic position using gene order data, we used constrained trees based on 41 mitochondrial genomes including 12 echinoderms, 2 hemichordates, 6 urochordates, 3 ecdysozoans and 17 lophotrochozoans (additional files 3 and 4) and calculated breakpoint scores (the number of gene adjacencies) and inversion scores (the number of gene inversions) for 10 alternative placements of *Xenoturbella bocki *(additional file 5).

Based on gene order alone, the most parsimonious position for *Xenoturbella *is as a basal hemichordate (hypothesis X6, table 1). Placing *Xenoturbella *in this position results in an inversion median score of 398 (i.e. one needs to posit 398 separate inversions to explain the entire phylogeny with *Xenoturbella *as a basal hemichordate) and a breakpoint score of 580 (i.e. there are 580 instances in the phylogeny in which a descendant sequence does not share a gene pairing with its parent sequence). It is of note that another placement of *Xenoturbella *(as a basal bilaterian/protostome/deuterostome) receives the same inversion score. However, the inversion score is calculated under the assumption that inversions are the only process underlying mitochondrial genome rearrangements, which is unlikely to be true. Given that the breakpoint score does not rely on this assumption, it is likely to represent a slightly better estimate of distances between mitochondrial genomes [41]. It seems sensible therefore to place more weight on the breakpoint than on the inversion score. On this basis, we conclude that gene order evidence favours the phylogenetic placement of *Xenoturbella *as sister group of the hemichordates.

**Table 1:** Inversion median and breakpoint scores for different hypotheses about the phylogenetic placement of *Xenoturbella*.

Abbreviation	Hypotheses	Inversion Median	Breakpoint
X1	basal vertebrate	401	597
X2	basal cephalochordate	403	602
X3	basal chordate	406	607
X4	basal ambulacrarian	403	602
X5	basal echinoderm	402	593
**X6**	**Basal hemichordate**	**398**	**580**
X7	basal bilaterian/protostome/deuterostome	398	587
X8	mollusc, order Nuculoida	406	608
X9	basal lophotrochozoan	404	600
X10	basal ecdysozoan	407	607

Lower scores are better. Best scores are shown in bold.

### Analysis of non-coding regions and transcription initiation sites

In vertebrates, each mitochondrial DNA strand (heavy strand and light strand) has its own control region, which forms a stable stem-loop structure [4]. The main portion of mitochondrial DNA involved in transcription and replication of the heavy (H) strand in vertebrates is called the D-loop region and has been well characterised [24,27]. The vertebrate D-loop region typically lies between *trnP *and *trnF *and is divided into three main domains: Extended termination-associated sequences (ETAS), central domain (CD) and conserved sequence block (CSB), each of them carrying particular conserved functional motifs and often repeats. The ETAS region may contain a number of repeats containing termination associated sequences (TAS), TACAT elements, associated with premature termination of the replication cycle [42].

The origin of replication of the light strand (O_L_) is normally between *trnN *and *trnC *in vertebrates and it also forms a typical stem loop structure. In the stem region of the O_L_, the trinucleotide 5'-CGG-3' is considered the initiation site for light strand replication in the mouse [43]. The loop is usually C/T-rich as it seems that pyrimidine stretches are required for the initiation of L-strand replication by an RNA primase [42].

The putative *Xenoturbella bocki *control region is a 334 bp stretch between *trnL *and -*nd6*. The *Xenoturbella *control region is noticeably A+T rich (79%). As a way of comparison, 3rd codon positions of the protein coding genes, which have low structural constraints due to the redundancy of the genetic code and tend to reflect the mutational pressure acting on the genome, are clearly less A+T rich (68%). This implies a weak structural constraint acting on the control region, even lower than that acting on the 3rd codon position or even a positive selection for A+T. This may suggest that only a small fraction of the control region (e.g. a few residues) is under stabilising selective positive pressure.

Another intriguing aspect is that the *Xenoturbella *control region is not strand asymmetric with respect to G and C (GC skew 0.03) while the rest of the genome is clearly GC skewed (-0.63 GC skew calculated on 3rd codon position, -0.26 on the whole genome). Mitochondrial genomes usually tend to become asymmetric towards G and C because one strand remains single stranded for longer compared to the other [44]. The lack of asymmetry in the control region of *Xenoturbella *may suggest that this region is permanently maintained in a double stranded condition. Vertebrates, hemichordates and crinoids show a significant GC asymmetry in their control region [45], while basal chordates such as cephalochordates and most of the Ambulacraria, groups that have shorter control regions than in *Xenoturbella*, have a very low GC asymmetry (data not shown). The *Xenoturbella *control region contains a number of other interesting features.

**Figure 2:**
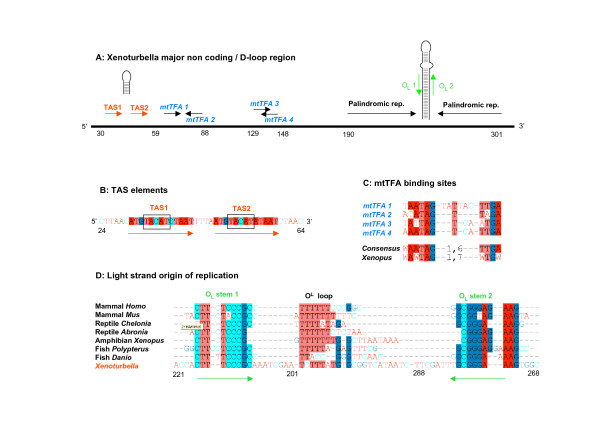
**Structure and putative major features of the *Xenoturbella *control region**. (A) General organization of the *Xenoturbella *control region (represented as a black line). Structural characters, putative elements and repeats are shown above the line, and their relative position is shown below. A repeated sequence that contains TAS elements is present at the 5' end, close to *trnL*. This sequence (represented by two red arrows) is able to form a short stem loop. In the central part there are 2 pairs of sequences corresponding to binding sites for the transcription factor mtTFA. The 3' region, close to the *nad6 *gene, is characterized by two long palindromic repeats that can form a stable, but imperfect (note the asymmetric internal loop), stem loop structure. A short region in the stem (green arrows) shows a high degree of similarity to the vertebrate Light strand origin of replication (O_L_). (B) The segment containing TAS elements (TACAT) consists of an almost exact 13 nucleotide repeat (red arrows). (C) Alignment of the 4 *Xenoturbella *mtTFA binding sites. The consensus sequence for the 4 *Xenoturbella *binding sites is compared to the vertebrate consensus *(Xenopus laevis)*. (D) Alignment of a portion of the *Xenoturbella *3' stem-loop to O_L _stem-loop sequences from a variety of vertebrate species. The two stems in *Xenoturbella *were inverted before alignment.

The 5' end (adjacent to *trnL*) and the central part of the control region are particularly AT enriched and characterised by a very low complexity. However, between positions 30 to 59 we could identify two 13 nucleotide tandem repeat sequences (figure 2B). Interestingly, these sequences contain putative TAS elements. The similarity with vertebrate TAS elements is rather convincing if we consider that these elements are generally found in repeated sequences and are only located at one extremity of the D-loop region in vertebrates (the 5' end, close to *trnP*). We have looked for the same TAS elements in other putative Ambulacrarian control regions, but we could not find any. A portion of this repeat is palindromic and capable of forming a stable stem-loop structure (-11,7 Kcal/m), which may help in the termination of replication [45].

We were also able to detect a putative binding site for mtTFA, the main mitochondrial transcription factor in vertebrates. The binding site consists of a pair of palindromic sequences that are located between positions 59 (there is one nucleotide overlap with one of the TAS containing repeats) and 91. The consensus for the 2 *Xenoturbella *binding sites can be seen in figure 2C and is almost identical to the consensus found in *Xenopus *[46]. It is also possible to identify another pair of putative binding sites at position 130 which partially overlap, rather than being separated by a 0–7 nucleotide gap, as expected in a binding site for multimeric DNA binding factors. However these putative binding sites have to be considered highly significant, as considering the *Xenoturbella *D-loop region nucleotide frequencies, we expect to find one every 50,000 nucleotides by chance.

Perhaps the most striking feature of the putative *Xenoturbella *D-loop is the presence of two 41 nucleotide long palindromic repeats (position 190 and 253). These repeats are almost identical and are able to form a very stable (-51 Kcal/m) stem loop structure, characterised by a short T-rich internal loop (figure 2A). We were unable to find stem loops of this size in other ambulacrarian or chordate D-loop regions. However, a portion of this stem has a striking similarity with the stem region of the light strand origin of replication (OL) of vertebrates. In figure 2D we have aligned the OL for different vertebrate species from human to an actinopterigyan fish (to our knowledge the most distant chordate from humans for which a light strand origin of replication was reported; [47]). As can be seen, a portion of *Xenoturbella *stem is clearly alignable with the OL stem of vertebrates, although we have had to reverse the sequence of the stems before alignment. Statistically, the consensus sequence for the vertebrate OL stem is expected to be found once in every million base pairs in the *Xenoturbella *genome; consequently it is unlikely that this similarity is a coincidence. We have looked for the consensus of this alignment in other ambulacrarians, urochordates and cephalochordates but could not find any similar sequence.

We were unable to identify any conserved sequence blocks (CSB) in the *Xenoturbella *non-coding region. These have been found in basal cephalochordates [48] but not in urochordates [49]. We also could not find any significant G stretches followed by AT dinucleotides as reported to be present in some echinoderms and in hemichordates [21].

We have detected features that are typical of vertebrate mitochondrial control regions, such as two putative termination associated sequences (TAS), binding sites for mtTFA and a significant similarity between the stem of the major stem loop structure of *Xenoturbella *and the stem of the vertebrates O_L_. These findings imply that the replication and transcription system of *Xenoturbella *may be more similar to the one found in chordates than to that of Ambulacraria, suggesting that commonalities of *Xenoturbella *and vertebrates may represent the ancestral state.

### Phylogenetic analyses

We have performed various phylogenetic analyses using the 13 concatenated mitochondrial proteins from 24 deuterostome species plus 7 outgroups. Perseke and colleagues reported that under certain analytical conditions *Xenoturbella *unexpectedly groups with the acoelomorph *Paratomella rubra *[16] among basal bilaterians and suggested that this may be correlated with the long branch leading to the acoelomorph. In order to evaluate this, and to test further any possible affinity of *Xenoturbella *with basal bilaterians we have analyzed the affinities of the acoelomorph in the presence and absence of the urochordates, which are characterised by accelerated substitution rates and therefore useful for the diagnosis of long branch artefacts (LBA). Our results show that in the absence of the long branched urochordates, the acoelomorph is basal to all other bilaterians (MrBayes/MtRev pp 100, tree not shown), while *Xenoturbella *is recovered within the deuterostomes (100). In the presence of the urochordates, the acoelomorph is strongly associated with the urochordates (MrBayes pp100, tree not shown) in a deuterostome clade, but not with *Xenoturbella*. These findings suggest that the acoelomorph is genuinely subject to LBA and consequently the reported relationship with *Xenoturbella *in a basal bilaterian position is likely to be the effect of an artefact. In the light of this, we have excluded the acoelomorph from subsequent analyses, but kept the problematic urochordates as an internal diagnostic of the ability of different models to fit the dataset accurately and avoid long branch artefacts.

We have run subsequent analyses of the mitochondrial dataset using optimal models of amino acid evolution. Using MrBayes and the mechanistic GTR model we analysed amino acids recoded into 4 functional categories, as this has been shown to be a helpful approach when dealing with problematic phylogenies ([50], see materials and methods for more details). We have also analyzed standard 20 character state amino acid sequences using two new empirical models of evolution, MtZoa and MtHydro, which we have implemented ourselves in MrBayes. The two models, in particular MtHydro, fit many mitochondrial datasets and in particular deuterostome datasets, better than MtREV (Rota-Stabelli O, Horner D, Telford MJ: MtHydro: a partitioned model for mitogenomics studies based on protein structural information, submitted), [31] (see materials and methods for more details). We have also used the CAT model, implemented in PhyloBayes [32], which accounts for across site heterogeneities in the amino-acid replacement process and CAT-BP, implemented in NHPhyloBayes [33]. These two models, in particular CAT-BP, have been shown to give an invaluable improvement in the case of compositionally heterogeneous datasets such as the mitogenomic one [32].

**Figure 3:**
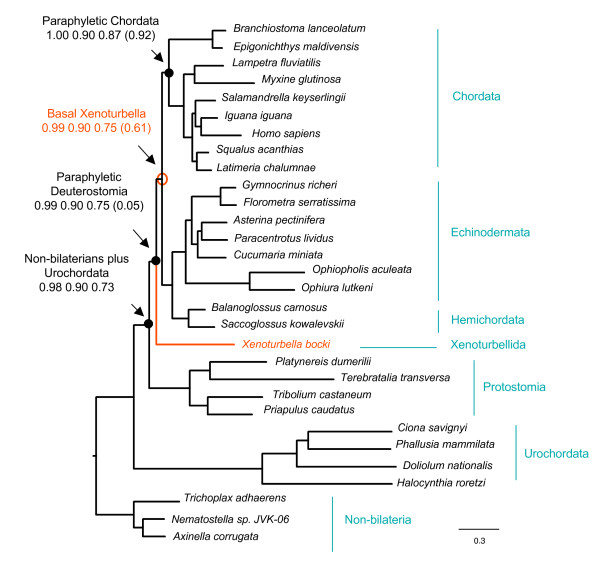
**Consensus tree from bayesian analyses using different amino acid models**. Topology and branch lengths correspond to the consensus tree from a bayesian analysis using the MtHydro model. Values at nodes are the posterior probabilities using the following models from left to right respectively: amino acid functional recoding, MtZoa, MtHydro and the CAT model (in brackets). All models place *Xenoturbella *as a basal deuterostome. Functional recoding, MtZoa and MtHydro support paraphyletic deuterostomes, due to LBA between the urochordates and the non-bilaterians. The CAT model supports monophyletic deuterostomes, although it groups the urochordates with the echinoderms. Note that the lower the support for paraphyletic deuterostomes, the lower the support for *Xenoturbella *as basal deuterostome.

Our results show that trees obtained using MrBayes consistently support a basal deuterostome position for *Xenoturbella *(see figure 3) in accordance with Perseke and colleagues [16]. This hypothesis is strongly supported by using the 4 amino acid functional recoding (99 pp), while, interestingly, using MtZoa (90 pp) and MtHydro (75 pp) the support decreases. In all the tree analyses the urochordates branch with the long branched outgroups, but support for this decreases from using the 4 functional categories (98 pp) to MtZoa (90 pp) and MtHydro (73 pp). We suggest that urochordates may be used here as an internal diagnostic of how the models fit the dataset. We suggest that as the model tends to escape a "wrong" urochordate position (with the outgroups, making chordates polyphyletic), the better the model fits the dataset and reduces the amount of false phylogenetic signal. Interestingly the decrease in support for urochordates+outgroup in the three models (98, 90, 73 respectively for functional recoding, MtZoa and MtHydro) is proportional to the decrease in support for a basal deuterostome position for *Xenoturbella *(99, 90, 75).

**Figure 4:**
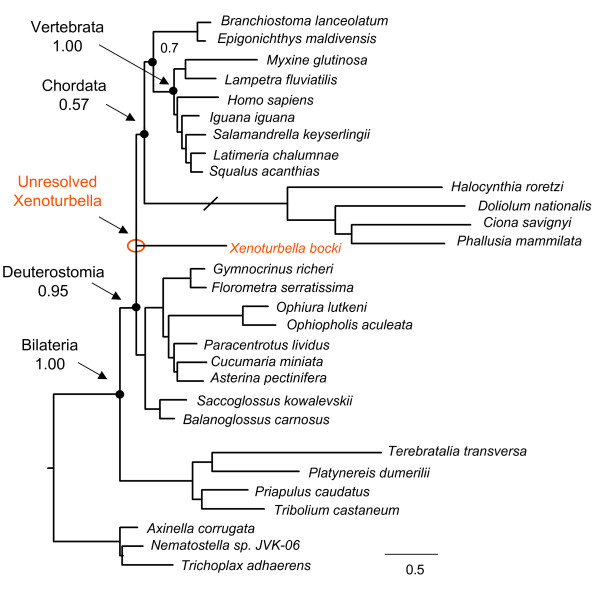
**Consensus tree from bayesian analysis using the CAT-BP model**. The model is able to recover the monophyly of Chordata, but does not resolve the position of *Xenoturbella *within monophyletic Deuterostomia. Branch length for the Urochordata has been halved.

Using PhyloBayes and modelling protein evolution with the CAT model [32], *Xenoturbella *is still recovered as a basal deuterostome, but with low support (61 pp) and the urochordates are recovered as deuterostomes with high support (95 pp), even if within paraphyletic echinoderms, rather than with the chordates. Using CAT-BP [33], which accounts for compositional heterogeneity within lineages, the urochordates are finally recovered as basal chordates, even if with tepid support (pp 57) (see figure 4). Interestingly, using CAT-BP the position of *Xenoturbella *is unresolved, implying that signal supporting a basal deuterostome position for *Xenoturbella *finally decreases to below 50%.

The ability of a model to handle problematic taxa, such as the urochordates, is a clear indication that this model fits the dataset well. The overall scenario of our tree searches suggests that the better the model is able to cope with the problematic urochordates, the weaker the support for *Xenoturbella *as basal deuterostome. Our tentative conclusion is that more adequate models recognise the apparent signal placing *Xenoturbella *in a basal deuterostome position as false, leaving the tree topology unresolved by mitochondrial gene sequences. We finally compared three competing tree topologies using the AU test and implementing the MtZoa model. Results indicate that the favoured tree topology is that of *Xenoturbella *as basal deuterostome (p value 0.808). However, a position as a basal Ambulacrarian (sister to hemichordates and echinoderms) is not rejected and has a p value of 0.247, much higher than the threshold for rejection that is 0.05. As a way of comparison, a basal chordate position for *Xenoturbella *is rejected at a level of 0.041.

Previous phylogenetic analyses based on the small ribosomal subunit [8] and large EST datasets [7,15] support a position of *Xenoturbella *as a basal Ambulacrarian. The basal deuterostome position obtained with the mitochondrial dataset using different models possibly reflects the compositional bias coming from mitochondrial genetic code changes in the lineage leading to Ambulacraria. When these amino acids (M, I, N, K) with a different genetic code in ambulacrarian mitochondrial genomes are excluded from the analyses, *Xenoturbella *is recovered as a basal ambulacrarian [7].

## Conclusion

Comparative analyses of the mitochondrial genome structure of *Xenoturbella bocki *with those of other deuterostomes and bilaterians have revealed a number of characteristics linking it to the deuterostome clade. Although the *Xenoturbella *mitochondrial gene order is very conserved and resembles the gene order found in the deuterostome ancestor (and possibly the bilaterian ancestor), breakpoint and inversion scores have revealed a closer similarity to the hemichordates. Additionally, analyses of transcription and replication initiation sites in the control region show a number of domains typical of vertebrate mitochondrial control regions. Together these results suggest that even though *Xenoturbella *seems morphologically derived within the deuterostomes (due to a dramatically simplified body plan), many structural and organisational aspects of its mitochondrial genome appear to be ancestral for the deuterostome group.

Finally, while standard phylogenetic analyses of the mitochondrial sequences support a basal deuterostome placement, the use of more sophisticated models of sequence evolution, specifically designed to counteract obvious systematic biases such as the known changes in genetic code amongst the deuterostomes, show decreasing support for this placement. We conclude that the mitochondrial genome sequence cannot be used to contradict the results derived from the much more comprehensive datasets of nuclear proteins and ribosomal RNAs which consistently show that *Xenoturbella *is the sister group of the Ambulacraria.

## Methods

### Mitochondrial genome sequencing

The complete mitochondrial genome of *Xenoturbella bocki *was obtained using a combination of conventional PCR and long PCR to amplify overlapping fragments spanning the whole mitochondrial genome. Specimens were collected by dredging in soft muddy sediment at around 60 m depth in the Gullmarsfjord close to Kristineberg Marine Station, Sweden. Individual live specimens were starved for 3 weeks and used whole for DNA extraction. The tissue was ground in homogenisation buffer using a pellet pestle. Nuclei were removed by centrifuging 3 × 10 minutes at 1500 *g *at 4°C. Mitochondria, suspended in the supernatant, were pelleted by centrifugation at 10,000 *g *for 10 minutes at 4°C and mtDNA extracted using the Wizard Minipreps DNA Purification System (Promega). Initially, cox3, nd4, rrnL and rrnS were amplified by conventional PCR using degenerate primers designed from published deuterostome mitochondrial sequence alignments (additional file 6). These PCR products were cloned and sequenced and the data were used in conjunction with already published cox1 and cox2 sequences to design specific Xenoturbella primers to amplify large overlapping regions of the Xenoturbella mitochondrial genome using long PCR. All fragments were cloned and sequenced as described below. For amplifications of 2 kb or less, PCR products were amplified using Titanium Taq DNA polymerase (Clontech), in a G-storm thermal cycler (Gene Technologies Ltd).

PCR program: 1 cycle: 94°C, 2 minutes. 30 cycles: 94°C, 30 sec; 50°C, 60 sec; 72°C 90 sec. 1 cycle: 72°C, 10 minutes. PCR products were purified using MinElute gel extraction kit (Quiagen), cloned in TOPO TA vector (Invitrogen), and sequenced using fluorescent dye terminator (Applied Biosystems). The sequencing reactions were run on an Applied Biosystems 377 automated sequencer. Long PCRs (GeneAmp XL PCR kit, Applied Biosystems) were carried out using specific primers in the *cox1, cox2, cox3, nd4, rrnL *and *rrnS *genes. Fragment sizes and primer sequences can be found in additional file 6. PCR program: 1 cycle, denaturation 94°C, 1 minute. 30 cycles, anneal/extend: 94°C, 30 sec; 65°C, 10 min. 1 cycle, final extension: 72°C, 10 minutes. PCR products (additional file 6) were cloned using the TOPO XL PCR cloning kit (Invitrogen). Plasmids were checked for inserts by restriction digest at the multiple cloning site. Transposons insertion (EZ-Tn5 <TET-1>, Epicentre) was carried out for 2 hours at 37°C. Colonies were grown on LB agar containing Tetracycline and Kanamycin, to select for tranposon insertions within the insert. Clones containing random transposon insertions were sequenced in both orientations, using priming sites in the transposon (TET-1 FP-1: 5'-GGGTGCGCATGATCCTCTAGAGT-3' and TET-1 RP-1: 5'-TAAATTGCACTGAAATCTAGAAATA-3').

### Annotation and alignment of mtDNA

Sequences were edited and overlapping contigs assembled using Lasergene V.7 (DNASTAR, Lasergene). Gene sequences were identified using BLAST and by alignment with mitochondrial sequences from other species (ClustalX). The genome sequence was deposited in GenBank, accession number DQ832701.

### Xenoturbella mitochondrial genetic code determination

This was done as described in Telford *et al*. [38].

### Breakpoint analysis

A phylogeny of 41 metazoan species (additional files 3 and 4) with sequenced mitochondrial genomes was constructed based on recent molecular phylogenetic studies [19,51-56]. Mitochondrial gene orders (including tRNAs) were downloaded from the OGRE database , and more recently published gene orders which were not available on the OGRE database were taken from NCBI . Accession numbers can be found in additional file 3.

Species whose mitochondrial genomes have divergent gene content (different from the 13 protein coding and 24 structural RNA genes typical of bilaterian animals) were not included in the analysis, due to limitations of the method of inferring ancestral gene orders [57,58]. Furthermore, in order to simplify the analysis, the *Homo sapiens *gene order was taken to be representative of the highly conserved vertebrate gene order, and the *Limulus polyphemus *gene order was taken to be representative of the highly conserved arthropod gene order [59]. For all other taxa, the maximum possible number of genome sequences were included in the analysis, given available genome and phylogenetic information. A summary of the assumed phylogeny is shown in additional file 4.

Inversion median and breakpoint scores were then calculated using the Circal package [57,58] for 10 different phylogenetic trees, in each of which the *Xenoturbella *sequence was placed in a different position on the phylogeny (additional file 5). In this way, it was possible to assess the best supported position of *Xenoturbella *within a phylogeny of the Metazoa based on gene order evidence.

### Control region

We searched for repeats in the non coding regions with TRF [60] and REPFIND [61]. We used Transfac [62], PATSEARCH [63] and RERNA  to look for conserved patterns and binding sites. We calculated the expected occurrence of a certain nucleotide motifs in the *Xenoturbella *control region by simple multiplication of the expected frequency of each nucleotide or class of nucleotide, using the nucleotide frequency of the *Xenoturbella *major control region.

### Phylogenetic analyses and tests of alternative hypotheses

We retrieved the 13 protein sequences of 30 metazoan species from the OGRe. database [22], GenBank accession numbers: NC_001912, NC_006465, NC_001131, NC_002639, NC_008082, NC_002793, NC_001807, NC_002012, NC_001804, NC_007689, NC_001878, NC_001627, NC_005929, NC_001572, NC_005334, NC_005930, NC_001887, NC_007438, NC_008556, NC_000931, NC_003086, NC_003081, NC_008557, NC_004570, NC_009833, NC_006627, NC_002177, NC_008164, NC_006894, NC_008151. We aligned protein sequences with MUSCLE [64]. The concatenated alignment was refined by hand and poorly conserved or ambiguously aligned codons were excluded from further analyses. Application of Gblocks [65] at default settings lead to a final alignment of 2572 reliably aligned amino acids. We have selected 7 outgroup species from the protostomes and non bilaterian metazoans on the basis of reduced compositional divergence, in accordance with the optimal outgroup selection of Rota-Stabelli and Telford [66]. We have also added available sequences from the Acoelomorph *Paratomella rubra *(AY228758).

Most of the tree searches were performed with the program MrBayes v3.1 under different models of evolution. We have used two new empirical models of mitochondrial amino acid evolution, MtZoa and MtHydro and recompiled MrBayes substituting these new models for existing ones [31], (Rota-Stabelli O, Horner D, Telford MJ: MtHydro: a partitioned model for mitogenomics studies based on protein structural information, submitted). While MtREV is based on the analysis of vertebrate species only, both MtZoa and MtHydro models have been estimated from the alignment of the 13 mitochondrial proteins from a large sample of 100 Metazoans, which contains many non-vertebrates deuterostomes, meaning that MtZoa and MtHydro may be a better estimator of animal mitochondrial evolution. Both models have been estimated using the maximum likelihood approach implemented in PAML [67] and assuming reversibility of the replacement process. MtHydro is based on the crystallographic structure of cytochrome oxidase b and complex IV subunits and bioinformatic prediction, which have been used to generate two partitions: one containing hydrophobic alpha-helices and one containing the remaining mostly hydrophilic sites. From the two partitions we have estimated two corresponding substitution matrices, which are characterized by different amino acid frequencies and replacement rates and are intended to be used simultaneously as a single model when modelling corresponding partitioned datasets (Rota-Stabelli O, Horner D, Telford MJ: MtHydro: a partitioned model for mitogenomics studies based on protein structural information, submitted). MtZOA is a general empirical model which implies among-site homogeneity, as in MtRev: all sites of the alignment are treated equally and modelled using the same replacement matrix and stationary frequencies [31].

Tests of model fit using AIC and BIC methods show that the two models, in particular MtHydro, are preferable to existing empirical models such as MtREV, when a dataset of deuterostomes is analyzed. We have also recoded amino acids into 4 functional groups according to amino acid chemical and physical properties. We partially based the groupings on the six classical groups of amino acids [50], and reduced them to four classes in order to analyze them under the 4 × 4 GTR model implemented in MrBayes 3.1. Groupings were as follows: group 1 (aromatic): FYW, group 2 (hydrophobic): VMLIC, group 3 (small): AGPSTNQ and group 4 (charged): HKRDE. It has been shown that functional recording is an effective means of decreasing nonphylogenetic signal and may help to infer phylogeny in the case of problematic taxa [50].

For all the MrBayes analyses, we used an invariable plus eight gamma distribution for modelling sites rate categories. We have run 2 MCMC chains for each dataset and stopped when the standard deviation of split frequencies between the two runs was less than 0.02.

Additional tree searches were made with the program PhyloBayes [32] and NhPhyloBayes [33], which respectively implement the models CAT and CAT-BP. While both models assigned different sites of the alignment to different classes characterised by different equilibrium frequencies [32], CAT-BP also accounted for compositional heterogeneity among lineages, which is an invaluable improvement in the case of compositionally heterogenic mitochondrial sequences [66]. We ran two independent chains in both PhyloBayes and NH-PhyloBayes. We stopped the chains when the largest discrepancy across bipartitions reached 0.15 in PhyloBayes. Statistical tests of the robustness of tree topologies have been evaluated with the Approximately Unbiased (AU) test of Shimodaira [68] and were performed on the nucleotide dataset using the software CONSEL [68] with site likelihood scores exported from PAML [69] and calculated using the MtZoa model and the tree topologies from MrBayes trees searches.

The MtZoa and MtHydro matrices are available at: . Additional information can be obtained from the authors upon request.

## Abbreviations

cox1, cox2, cox3: cytochrome oxidase subunit I, II, and III protein genes; cob: cytochrome b gene; atp6, atp8: ATP synthase subunit 6 and 8 genes; nad1, nad2, nad3, nad4, nad4L, nad5, nad6: NADH dehydrogenase subunit 1–6, 4L genes; *rrnS *and *rrnL*: small and large ribosomal RNA genes; *trnA*, *trnC*, *trnD*, *trnE*, *trnF*, *trnG*, *trnH*, *trnI*, *trnK*, *trnL1*, *trnL2*, *trnM*, *trnN*, *trnP*, *trnQ*, *trnR*, *trnS1*, *trnS2*, *trnT*, *trnV*, *trnW*, *trnY*: transfer RNA genes, designated by the one-letter code for the specified amino acid; '-': designates a gene on the opposite strand, eg. -nd6; O_L_: Light strand origin of replication; LBA: Long branch attraction; PP: Posterior probability

## Authors' contributions

SJB sequenced the mitochondrial genome, analyzed the gene order data and led the write up. MJT analyzed the codon usage, RL ran the breakpoint analysis and ORS analyzed the control region. MJT and ORS developed the MtZoa and MtHydro models and ORS ran the phylogenetic analyses. All authors read and approved the final manuscript.

## Supplementary Material

Additional file 1 **Annotation of the *Xenoturbella bocki *mitochondrial genome**. Position, orientation, size, start and stop codons of protein coding and tRNA genes as well as gaps along the circular mitochondrial genome of *Xenoturbella bocki*. Click here for file

Additional file 2 **Genetic code and codon usage of all protein coding genes from the mitochondrial genome of *Xenoturbella bocki***. Click here for file

Additional file 3 **Species used in the breakpoint and inversion analyses**. Genbank accession numbers of the mitochondrial genomes used in breakpoint and inversion analyses. Click here for file

Additional file 4 **Assumed phylogeny of Bilateria**. Assumed phylogeny of Bilateria (12 echinoderms, 2 hemichordates, 6 urochordates, 3 ecdysozoans and 17 lophotrochozoans for which mitochondrial genomes are available), used in the breakpoint and inversion analyses. The phylogeny was constructed from a number of different sources (see main text). Click here for file

Additional file 5 **Hypotheses tested for the phylogenetic position of *Xenoturbella bocki***. Different hypotheses were tested by using breakpoint and inversion scores for the phylogenetic affiliation of *Xenoturbella*: X1 – basal vertebrate; X2 – basal cephalochordate; X3 – basal chordate; X4 – basal ambulacrarian; X5 – basal echinoderm; X6 – basal hemichordate; X7 – basal bilaterian/protostome/deuterostome (unresolved due to the lack of an available root for the tree); X8 – mollucs, order Nuculoida; X9 – basal lophotrochozoan; X10 – basal ecdysozoan. Click here for file

Additional file 6 **Primer pairs and fragment sizes obtained in mitochondrial genome amplification**. Click here for file
