# Supplementary material for: The mitochondrial genome structure of Xenoturbella bocki (phylum Xenoturbellida) is ancestral within the deuterostomes
Source: BMC Evol Biol. 2009 May 18;9:107. doi: 10.1186/1471-2148-9-107 (PMC2697986; doi:10.1186/1471-2148-9-107)
Supplement: Additional file 1 — Annotation of the Xenoturbella bocki mitochondrial genome. Position, orientation, size, start and stop codons of protein coding and tRNA genes as well as gaps along the circular mitochondrial genome of Xenoturbella bocki. [file 1471-2148-9-107-S1.doc]

| Annotation of the *Xenoturbella bocki* mitochondrial genome | | | | | | | |
| --- | --- | --- | --- | --- | --- | --- | --- |
| gene | strand | position number | Size (bp) | Size (aa) | Start codon | Stop codon | Intergenic nucleotides |
| *cox1* | + | 1-1551 | 1551 | 516 | ATG | TAA | -1 |
| *trnS* | - | 1550-1615 | 66 |  |  |  | 0 |
| *trnD* | + | 1616-1683 | 68 |  |  |  | 0 |
| *cox2* | + | 1684-2362 | 678 | 226 | GTG | T | 0 |
| *trnK* | + | 2363-2430 | 68 |  |  |  | 1 |
| *atp8* | + | 2432-2593 | 162 | 53 | ATG | TAA | -18 |
| *atp6* | + | 2575-3275 | 702 | 233 | ATG | TA | 0 |
| *cox3* | + | 3276-4063 | 789 | 262 | ATG | TA | 0 |
| *trnG* | + | 4064-4126 | 63 |  |  |  | 0 |
| *nad3* | + | 4127-4475 | 350 | 116 | ATG | T | 0 |
| *trnR* | + | 4476-4539 | 64 |  |  |  | 0 |
| *nad4L* | + | 4540-4833 | 294 | 97 | ATG | TAA | 0 |
| *nad4* | + | 4827-6196 | 1371 | 456 | ATG | TA | -6 |
| *trnH* | + | 6197-6262 | 66 |  |  |  | 0 |
| *trnS2* | + | 6256-6325 | 70 |  |  |  | -12 |
| *nad5* | + | 6313-8055 | 1743 | 580 | ATG | TAA | 3 |
| *cytB* | + | 8059-9201 | 1143 | 380 | ATG | TAA | 2 |
| *trnP* | + | 9204-9271 | 68 |  |  |  | 4 |
| *trnF* | + | 9276-9341 | 66 |  |  |  | 0 |
| *rrnS* | + | 9342-10106 | 765 |  |  |  | 0 |
| *trnV* | + | 10107-10172 | 66 |  |  |  | 1 |
| *rrnL* | + | 10174-11503 | 1330 |  |  |  | 0 |
| *trnT* | + | 11504-11571 | 68 |  |  |  | 0 |
| *trnL* | + | 11572-11639 | 68 |  |  |  | 1 |
| *trnL* | + | 11641-11705 | 65 |  |  |  | 0 |
| *CR* |  | 11706-12040 | 334 |  |  |  | 0 |
| *nad6* | - | 12041-12553 | 513 | 170 | ATG | TAA | 0 |
| *trnE* | + | 12554-12620 | 67 |  |  |  | 40 |
| *nad1* | + | 12661-13620 | 960 | 319 | ATG | TAA | 1 |
| *trnI* | + | 13622-13689 | 68 |  |  |  | 0 |
| *trnQ* | - | 13690-13752 | 63 |  |  |  | 1 |
| *trnM* | + | 13754-13820 | 67 |  |  |  | 0 |
| *nad2* | + | 13821-14891 | 1071 | 356 | ATG | TAA | 10 |
| *trnN* | - | 14902-14964 | 63 |  |  |  | 0 |
| *trnW* | + | 14964-15028 | 65 |  |  |  | 0 |
| *trnC* | - | 15029-15091 | 63 |  |  |  | 1 |
| *trnA* | - | 15093-15158 | 66 |  |  |  | 10 |
| *trnY* | - | 15169-15231 | 63 |  |  |  |  |
