# Supplementary material for: The mitochondrial genome structure of Xenoturbella bocki (phylum Xenoturbellida) is ancestral within the deuterostomes
Source: BMC Evol Biol. 2009 May 18;9:107. doi: 10.1186/1471-2148-9-107 (PMC2697986; doi:10.1186/1471-2148-9-107)
Supplement: Additional file 2 — Genetic code and codon usage of all protein coding genes from the mitochondrial genome of Xenoturbella bocki. [file 1471-2148-9-107-S2.doc]

| Genetic code and codon usage of all protein coding genes from the mitochondrial genome of *Xenoturbella bocki* | | | | | | | |
| --- | --- | --- | --- | --- | --- | --- | --- |
| Codon | % | Codon | % | Codon | % | Codon | % |
| AAA Lys | 5.05 | CCA Pro | 1.52 | GGA Gly | 2.72 | TTA Leu | 5.2 |
| AAC Asn | 2.62 | CCC Pro | 1.49 | GGC Gly | 0.71 | TTC Phe | 3.19 |
| AAG Lys | 0.26 | CCG Pro | 0.37 | GGG Gly | 0.84 | TTG Leu | 0.58 |
| AAT Asn | 1.62 | CCT Pro | 1.2 | GGT Gly | 0.76 | TTT Phe | 2.96 |
| ACA Thr | 4.45 | CGA Arg | 1.2 | GTA Val | 2.69 |  |  |
| ACC Thr | 2.07 | CGC Arg | 0.1 | GTC Val | 0.6 |  |  |
| ACG Thr | 0.16 | CGG Arg | 0.05 | GTG Val | 0.73 |  |  |
| ACT Thr | 1.78 | CGT Arg | 0.26 | GTT Val | 0.99 |  |  |
| AGA | 0 | CTA Leu | 4.18 | TAA End | 0.26 |  |  |
| AGC Ser | 0.94 | CTC Leu | 1.31 | TAC Tyr | 1.8 |  |  |
| AGG | 0 | CTG Leu | 0.37 | TAG End | 0.03 |  |  |
| AGT Ser | 0.81 | CTT Leu | 1.49 | TAT Tyr | 0.97 |  |  |
| ATA Met | 7.4 | GAA Glu | 1.73 | TCA Ser | 2.64 |  |  |
| ATC Ile | 4.24 | GAC Asp | 0.68 | TCC Ser | 1.49 |  |  |
| ATG Met | 1.02 | GAG Glu | 0.65 | TCG Ser | 0.31 |  |  |
| ATT Ile | 4.34 | GAT Asp | 0.92 | TCT Ser | 1.78 |  |  |
| CAA Gln | 1.86 | GCA Ala | 2.82 | TGA Trp | 2.33 |  |  |
| CAC His | 1.31 | GCC Ala | 2.2 | TGC Cys | 0.34 |  |  |
| CAG Gln | 0.18 | GCG Ala | 0.16 | TGG Trp | 0.16 |  |  |
| CAT His | 0.89 | GCT Ala | 1.94 | TGT Cys | 0.31 |  |  |
