# Supplementary material for: The mitochondrial genome structure of Xenoturbella bocki (phylum Xenoturbellida) is ancestral within the deuterostomes
Source: BMC Evol Biol. 2009 May 18;9:107. doi: 10.1186/1471-2148-9-107 (PMC2697986; doi:10.1186/1471-2148-9-107)
Supplement: Additional file 3 — Species used in the breakpoint and inversion analyses. Genbank accession numbers of the mitochondrial genomes used in breakpoint and inversion analyses. [file 1471-2148-9-107-S3.doc]

| Accession numbers for the mitochondrial genomes used in the breakpoint analysis. | |
| --- | --- |
| Genbank Accession | Species |
| NC10181 | Strongylocentrotus purpuratus |
| NC10421 | *Paracentrotus lividus* |
| NC11617 | *Arbacia lixula* |
| NC17741 | *Cucumaria miniata* |
| NC17538 | *Ophiopholis aculeata* |
| NC17742 | *Ophiura lutkeni* |
| NC19207 | *Acanthaster brevispinus* |
| NC19206 | *Acanthaster planci* |
| NC10513 | *Patiria pectinifera* |
| NC13342 | *Florometra serratissima* |
| NC19106 | *Phanogenia gracilis* |
| NC19105 | *Gymnocrinus richeri* |
| NC13475 | *Balanoglossus carnosus* |
| NC18788 | *Saccoglossus kowalevskii* |
| NC21332 | *Asymmetron inferum* |
| NC18070 | *Asymmetron lucayanum* |
| NC18071 | *Epigonichthys maldivensis* |
| NC16863 | *Branchiostoma belcheri* |
| NC13725 | *Branchiostoma lanceolatum* |
| NC15058 | *Branchiostoma floridae* |
| NC12188 | *Homo sapiens* |
| NC15758 | *Limulus polyphemus* |
| NC15606 | *Trichinella spiralis* |
| NC20047 | *Priapulus caudatus* |
| NC17636 | *Aplysia californica* |
| NC16728 | *Roboastra europaea* |
| NC17572 | *Biomphalaria glabrata* |
| NC11604 | *Albinaria caerulea* |
| NC12206 | *Cepaea nemoralis* |
| NC17648 | *Siphonodentalium lobatum* |
| NC17884 | *Graptacme eborea* |
| NC17539 | *Lampsilis ornata* |
| NC17982 | *Octopus vulgaris* |
| NC15475 | *Loligo bleekeri* |
| NC17752 | *Haliotis rubra* |
| NC10528 | *Katharina tunicata* |
| NC17956 | *Clymenella torquata* |
| NC19352 | *Orbinia latreillii* |
| NC10599 | *Lumbricus terrestris* |
| NC15124 | *Platynereis dumerilii* |
| NC18002 | *Urechis caupo* |
| NC20046 | *Xenoturbella bocki* |
