# Supplementary figures and images for: The mitochondrial genome structure of Xenoturbella bocki (phylum Xenoturbellida) is ancestral within the deuterostomes
Source: BMC Evol Biol. 2009 May 18;9:107. doi: 10.1186/1471-2148-9-107 (PMC2697986; doi:10.1186/1471-2148-9-107)

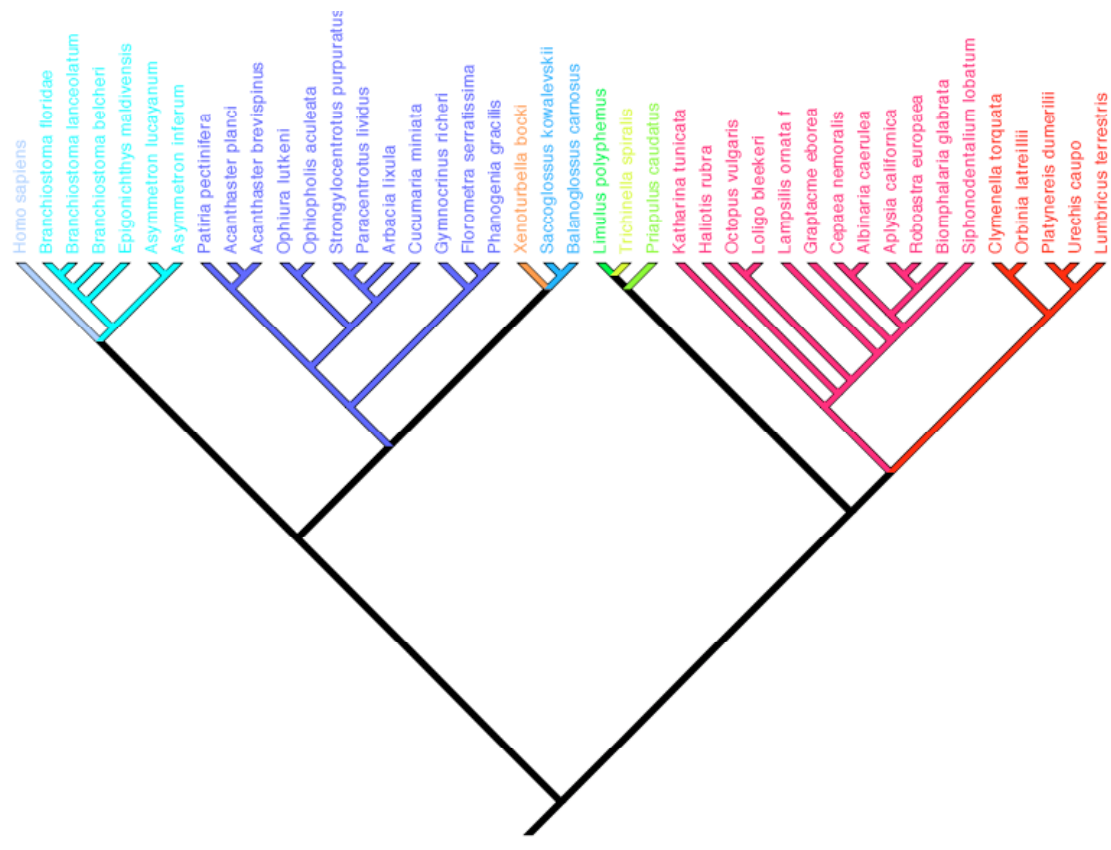

Supplement: Additional file 4 — Assumed phylogeny of Bilateria. Assumed phylogeny of Bilateria (12 echinoderms, 2 hemichordates, 6 urochordates, 3 ecdysozoans and 17 lophotrochozoans for which mitochondrial genomes are available), used in the breakpoint and inversion analyses. The phylogeny was constructed from a number of different sources (see main text). [file 1471-2148-9-107-S4.pdf]

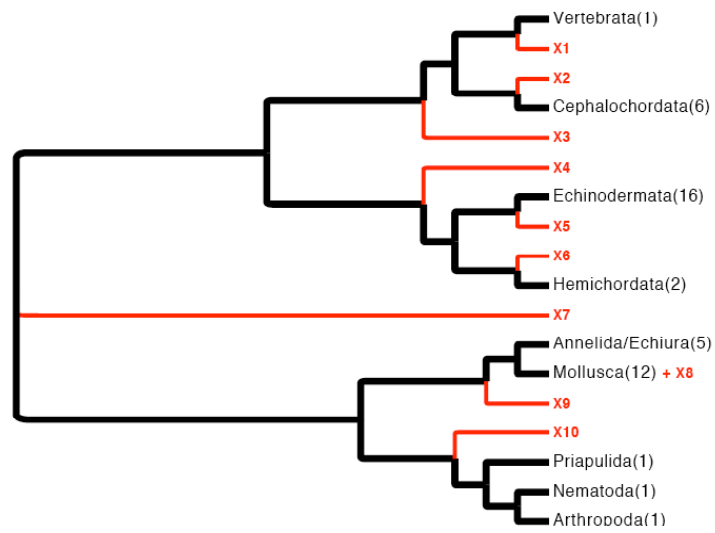

Supplement: Additional file 5 — Hypotheses tested for the phylogenetic position of Xenoturbella bocki. Different hypotheses were tested by using breakpoint and inversion scores for the phylogenetic affiliation of Xenoturbella: X1 – basal vertebrate; X2 – basal cephalochordate; X3 – basal chordate; X4 – basal ambulacrarian; X5 – basal echinoderm; X6 – basal hemichordate; X7 – basal bilaterian/protostome/deuterostome (unresolved due to the lack of an available root for the tree); X8 – mollucs, order Nuculoida; X9 – basal lophotrochozoan; X10 – basal ecdysozoan. [file 1471-2148-9-107-S5.pdf]
