# Supplementary material for: The mitochondrial genome structure of Xenoturbella bocki (phylum Xenoturbellida) is ancestral within the deuterostomes
Source: BMC Evol Biol. 2009 May 18;9:107. doi: 10.1186/1471-2148-9-107 (PMC2697986; doi:10.1186/1471-2148-9-107)
Supplement: Additional file 6 — Primer pairs and fragment sizes obtained in mitochondrial genome amplification. [file 1471-2148-9-107-S6.doc]

| Degenerate primers used in primary PCR amplifications | |
| --- | --- |
| cox3F | ATA GTT GAN CVH AGH CCH TG |
| cox3R2 | ACG TCT ACA AAR TGY CAR TAY CA |
| nd4F | CCC AAG GCH CAY GTN GAR GC |
| nd4R | ACT GAG GAG TAN GCD ATD A |
| lsuF | GGA TTA GAT ACC CYR YTA T |
| lsuR1  lsuR2 | GTG ACG GGC GRT GTG TRC G or TAC YDT GTT ACG ACT T |
| ssuF | TGA CCG TGC RAA GGT AGC |
| ssuR1  ssuR2 | CTG GAA CTC AGA TCA CGT A or ATC CAA CAT CGA GGT CGY AA |

| Specific primers used in long PCR | |
| --- | --- |
| cox1F | CTC AGA TTA TCC GGA TGC CTA TAG C |
| cox1R | CCA GTT CCA ACT CCA CTC TCT ACA C |
| cox3F | TCA TCA CTT CGG ATT CGA AGC AGC |
| cox3R | GGT GAG TGG AAT CAT AGG GCT AAT C |
| nd4F | GGT TGA AGA TAG GAG CTA TTC GTA GG |
| nd4R | AAG CAT AAT CTG CCT ACG ACA AAC TG |
| lsuF | AGG GTT TGT GAC CTC GAT GTT GG |
| lsuR | GGC TCT CGT TAT TCC TTT CAT TC |
| ssuF | GGC TCT AAA GTG CGC ACA GAT CG |
| ssuR | CAA GCT CCT CTA ATG GGA TAA AG |

| Primer combinations and long PCR fragment sizes | |
| --- | --- |
| cox1F-cox3R | 2 kb |
| cox3F-ssuR | 6 kb |
| cox3F-lsuR | 7 kb |
| lsuF-cox1R | 4.5 kb |
| lsuF-cox3R | 9 kb |
| ssuF-cox3R | 10 kb |
